# Association of MRI Measures With Disease Severity and Progression in Progressive Supranuclear Palsy

**DOI:** 10.3389/fneur.2020.603161

**Published:** 2020-11-12

**Authors:** Marina Picillo, Filomena Abate, Sara Ponticorvo, Maria Francesca Tepedino, Roberto Erro, Daniela Frosini, Eleonora Del Prete, Paolo Cecchi, Mirco Cosottini, Roberto Ceravolo, Gianfranco Di Salle, Francesco Di Salle, Fabrizio Esposito, Maria Teresa Pellecchia, Renzo Manara, Paolo Barone

**Affiliations:** ^1^Center for Neurodegenerative Diseases (CEMAND), Department of Medicine, Surgery and Dentistry, Neuroscience Section, University of Salerno, Fisciano, Italy; ^2^Department of Medicine, Surgery & Dentistry, Scuola Medica Salernitana, University of Salerno, Baronissi, Italy; ^3^Dipartimento di Medicina Clinica e Sperimentale Università di Pisa, Pisa, Italy; ^4^Dipartimento di Ricerca Traslazionale e delle Nuove Tecnologie in Medicina e Chirurgia, Università di Pisa, Pisa, Italy; ^5^Scuola Superiore Sant'Anna, Pisa, Italy; ^6^Department of Diagnostic Imaging, University Hospital A.O.U. OO.RR. San Giovanni di Dio e Ruggi D'Aragona, Scuola Medica Salernitana, Salerno, Italy; ^7^Department of Neurosciences, University of Padua, Padua, Italy

**Keywords:** progressive supranuclear palsy, imaging, disease severity, disease progression, milestones

## Abstract

**Objective:** To verify the association of midbrain-based MRI measures as well as cortical volumes with disease core features and progression in patients with Progressive Supranuclear Palsy (PSP).

**Methods:** Sixty-seven patients (52.2% with Richardson's syndrome) were included in the present analysis. Available midbrain-based MRI morphometric assessments as well as cortical lobar volumes were computed. Ocular, gait and postural involvement at the time of MRI was evaluated with the PSP rating scale. Specific milestones or death were used to estimate disease progression up to 72 months follow up. Hierarchical regression models and survival analysis were used for analyzing cross-sectional and longitudinal data, respectively.

**Results:** Multivariate models showed vertical supranuclear gaze palsy was associated with smaller midbrain area (OR: 0.02, 95% CI 0.00–0.175, *p* = 0.006). Cox regression adjusted for age, disease duration, and phenotype demonstrated that lower midbrain area (HR: 0.122, 95% CI 0.030–0.493, *p* = 0.003) and diameter (HR: 0.313, 95% CI 0.112–0.878, *p* = 0.027), higher MR Parkinsonism Index (HR: 6.162, 95% CI 1.790–21.209, *p* = 0.004) and larger third ventricle width (HR: 2.755, 95% CI 1.068–7.108, *p* = 0.036) were associated with higher risk of dependency on wheelchair.

**Conclusions:** Irrespective of disease features and other MRI parameters, reduced midbrain size is significantly associated with greater ocular motor dysfunction at the time of MRI and more rapid disease progression over follow up. This is the first comprehensive study to systematically assess the association of available midbrain-based MRI measures and cortical volumes with disease severity and progression in a large cohort of patients with PSP in a real-world setting.

## Introduction

Progressive Supranuclear Palsy (PSP) is a rare, rapidly progressive, neurodegenerative disease characterized by dysfunction in four core domains including ocular motor function, postural instability, akinesia, and cognition represented by a number of clinical features (1). The diverse combination of core clinical features is relevant for the attribution of the degree of diagnostic certainty as well as the clinical phenotype of disease (1). While PSP Richardson's syndrome (PSP-RS) is the most common clinical phenotype, other distinct variants, each featured by a specific predominant symptom, have been described (vPSP) (1). Irrespective of the phenotype, the presence of either slowing velocity of vertical saccades or vertical supranuclear gaze palsy (VSGP) is mandatory for the diagnosis of probable PSP (1).

To date, research studies on magnetic resonance imaging (MRI) measures in PSP have focused on supporting the clinical diagnosis of disease. Given the key role of midbrain in PSP-related pathological process, a number of midbrain-based MRI morphometric measures have shown adequate diagnostic accuracy in differentiating PSP-RS from healthy subjects and other parkinsonian disorders (2–7).

Conversely, few studies focused on the association between MRI measures and disease severity and progression in PSP. Evidence showed the MR Parkinsonism Index (MRPI) anticipated the development of VSGP in 11 out of 21 patients with PSP with predominant parkinsonism after a mean follow up of 28.5 months and a decreased midbrain-to-pons area ratio predicted shorter survival and earlier institutionalization in 51 patients fulfilling criteria for probable and possible PSP (8, 9). However, a comprehensive screening of the association of available midbrain-based MRI measures with disease severity and progression is lacking.

Demonstrating a relationship between MRI imaging markers and disease severity and progression in a real-world setting is pivotal to identify surrogate markers of disease to be used in the context of clinical trials evaluating disease-modifying treatments (10). Aim of the present study is to verify the association of midbrain-based MRI measures with disease core features and progression in patients with PSP. As most novel measures include a surrogate evaluation of other brain regions involved in the pathological process (e.g., frontal cortical atrophy in MRPI 2.0), we also analyzed the impact of cortical lobar atrophy on disease features and progression. In the cross-sectional phase of the study, MRI correlates of VSGP, severe postural instability and gait impairment were investigated. In the longitudinal phase, relationship between baseline MRI parameters and disease-specific milestones and survival was explored.

## Methods

### Patients and Clinical Evaluation

Sixty-seven patients with probable or possible PSP according to the Movement Disorder Society (MDS) criteria were included in the present analysis. Detailed information on enrollment and application of the PSP diagnostic criteria to determine disease phenotype is available elsewhere (7, 11, 12). Briefly, 78 PSP outpatients were enrolled from the Movement Disorders Centers of the University of Salerno and the University of Pisa between November 2015 and December 2018. Eleven patients were excluded because already presented one of the milestones at baseline (i.e., dementia) (see below). All had diagnosis of probable PSP but those with corticobasal predominant phenotype which—by definition—qualifies for possible PSP (1).

In a preliminary exploratory analysis, PSP rating scale considered as total score did not show any significant relationship with any MRI measure (data not shown). Thus, for the cross-sectional phase, severity of disease was evaluated according to specific items from the PSP rating scale scoring core features of the disease (13). As for ocular dysfunction, patients presenting a score >2 on both items 14 and 15 (i.e., saccadic amplitude reduced by more than 50% on the vertical plane) were considered affected by VSGP. As for postural instability, patients presenting a score >2 on item 27 (i.e., must be caught by the examiner on backward pull or requiring assistance to stand still) were deemed affected by severe postural instability. As for gait impairment, patients presenting a score >2 on item 26 (i.e., need for assistance all or almost all time or inability to walk) were considered affected by severe gait disturbance.

For the longitudinal phase, PSP rating scale was available only for a subset of patients (42) and no relationship was evident between change in PSP rating scale and baseline MRI measure in an exploratory analysis. Thus, the following milestones were selected to define disease progression: (1) dependence on wheelchair; (2) unintelligible speech; (3) dementia (i.e., cognitive impairment severe enough to significantly affect activities of daily living). These milestones have been selected because they are clinically relevant and represent the different domains of impairment of functioning in PSP (14, 15). All enrolled patients but 5 were re-evaluated after a mean (standard deviation) of 16.36 (11.51) months. Eleven patients (including 8 deceased) were not available for in-hospital follow up and a telephone assessment with caregiver was proposed. Thus, milestones were retrieved from medical records and/or with a semi-structured interview administered to the caregiver by telephone. Time to development of each milestone and death were calculated since baseline MRI (range: 1–72 months).

### MRI Imaging Protocol

Brain MRI was performed at the time of baseline evaluation. Eighty-two percent (55/67) of patients underwent 3T brain MRI with the same scanner (Skyra, Siemens, Erlangen, Germany); the remaining patients had MRI with different scanners (1.5 and 3T). On the 3T MRI scanner a volumetric 3D T1-weighted magnetization prepared rapid gradient echo (MPRAGE) sequence was acquired with the following parameters: repetition time = 2,400 ms, echo time = 2.25 ms, resolution = 1 × 1 × 1 mm^3^, matrix size = 256 × 256, 192 sagittal slices, anterior–posterior phase-encoding direction, generalized autocalibrating partially parallel acquisition (GRAPPA) factor of 2 in phase-encoding direction.

Sagittal partitions were obtained and multiplanar reconstructions were obtained in the conventional transverse and coronal planes.

### Morphometric Measurements

**Figure 1:**
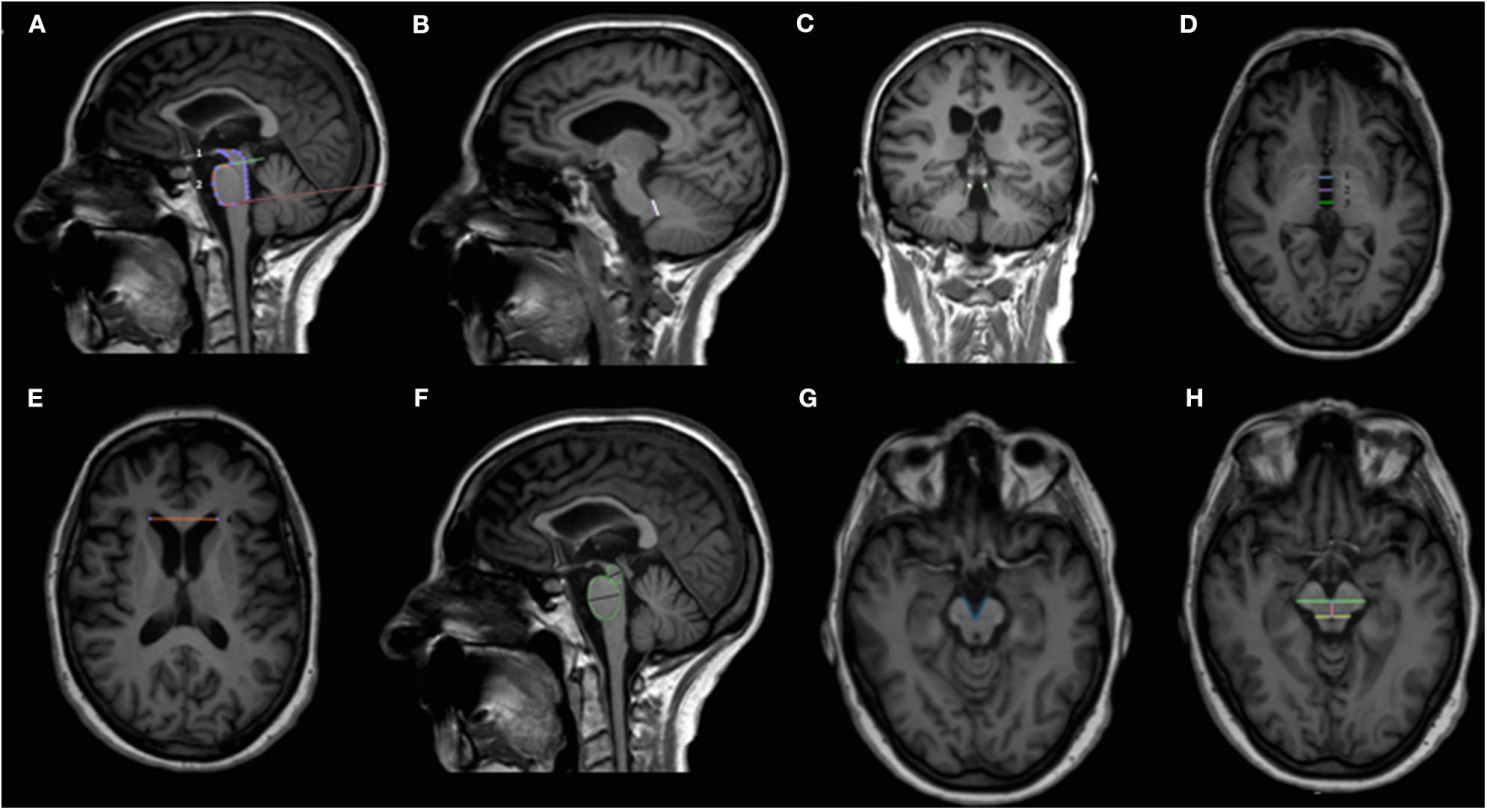
Overview of the midbrain-based measures computed. Sagittal **(A,B,F)**, coronal **(C)** and axial **(D,E,G,H)** T1-weighted volumetric MR images. Midsagittal midbrain area (1) and midsagittal pons area (2) used for calculating the pons-to-midbrain area ratio (P/M) are shown in **(A)**. Middle and superior cerebellar peduncles used to compute middle cerebellar peduncles to superior cerebellar peduncles ratio (MCP/SCP) are shown in **(B)** and **(C)**, respectively. Third ventricle measurements at the level of anterior and posterior commissures [third ventricle width derives from the mean of the anterior (1), medium (2), and posterior lines (3)] and frontal horns width are shown in **(D)** and **(E)**, respectively. MRPI is calculated with the following formula: (P/M) × (MCP/SCP); MRPI 2.0 is calculated with the following formula: MRPI × (third ventricle width/frontal horns width); P/M 2.0 is calculated with the following formula: (P/M) × (third ventricle width/frontal horns width). Midbrain and pons diameters obtained from midsagittal elliptical regions of interests are shown in **(F)**. Minor axes were used to calculate the pons-to-midbrain diameter ratio. The interpeduncular angle, calculated on a plane parallel to anterior commissure-posterior commissure line and right below the mammillary bodies, is shown in **(G)**. The length of midbrain tegmentum, measured as the distance between the interpeduncular fossa and the center of the aqueduct at the level of mammillary bodies, on a plane parallel to the anterior commissure-posterior commissure line is shown in **(H)**. MCP/SCP, middle cerebellar peduncles to superior cerebellar peduncles ratio; MR, magnetic resonance; MRPI, MR Parkinsonism index; MRPI 2.0, MR Parkinsonism index 2.0 version; P/M, pons-to-midbrain area ratio; P/M 2.0, pons-to-midbrain area ratio 2.0 version.

Midbrain-based measures were retrospectively calculated for all included patients and included midsagittal midbrain area, length of midbrain tegmentum, midbrain diameter, pons-to-midbrain diameter ratio, cerebral interpeduncolar angle, middle cerebellar peduncles to superior cerebellar peduncles ratio (MCP/SCP), pons-to midbrain area ratio (P/M), and MRPI as well as P/M 2.0 and MRPI 2.0 (MRPI and P/M multiplied by third ventricle width/frontal horns width ratio) (Figure 1) (3, 4, 7, 16–18). Given its relevance in PSP, both the latter include a surrogate measure of the frontal lobe volume (4, 10).

All midbrain measures were manually computed according to published methods by the same neuroradiologist (R.M.) with more than 15 years of experience in neurodegenerative diseases (7). Acceptable inter-rater agreement for manually computed measures between two different neuroradiologists as well as excellent agreement between manual and computerized MRPI have been already demonstrated elsewhere for the data considered in the present analysis (7).

Cortical volume was evaluated for a subset of 49 patients (73%). MPRAGE images were processed using FreeSurfer version 6.0 (https://surfer.nmr.mgh.harvard.edu/) using the standard structural image preprocessing and surface reconstruction pipeline via the “recon-all” command (for a detailed description of this procedure please see https://surfer.nmr.mgh.harvard.edu/fswiki/ReconAllTableStableV5.3) (19– 25). Preprocessed data were visually inspected to assure the quality of each reconstruction. Cortical volume was extracted for each region of the Desikan-Killiany cortical atlas and then regions were merged (and volumes were summed) to obtain one regional value for each cerebral lobe (frontal, parietal, occipital, temporal, and cingulate) (26).

### Standard Protocol Approvals, Registrations, and Patient Consent

The project was approved by the local Ethics Committees and each subject was included after signing the informed consent form.

### Statistical Analysis

Differences in variables between groups were computed with χ^2^ or Mann-Whitney test, as appropriate.

In line with the principles of multilevel modeling, hierarchical regression has been used for the present analysis (27). For the cross-sectional phase, multivariate logistic regression was implemented to explore imaging correlates of VSGP (non-VSGP = 0 vs. VSGP = 1), severe postural instability (non-severe postural instability = 0 vs. severe postural instability = 1) and severe gait impairment (non-severe gait impairment = 0 vs. sever gait impairment = 1). After adjusting for age and disease duration, the univariate relationship between each clinical outcome and each imaging parameter was examined. Any variables that had univariate associations with *p* values <0.10 were included in a multivariate model, also adjusting for disease phenotype (PSP-RS vs. vPSP). A backward selection approach was used to choose the best model. Variables were removed one at a time until all variables remaining in the model were significant at the 0.05 level. To avoid collinearity issues between individual MRI measures and midbrain ratios, if any individual measures were significant, they were considered in the multivariate model. The midbrain ratios were only considered in the multivariate model if neither of the individual components was significant. All odds ratio (OR, CI 95%) and *p*-values are from the backwards selection models.

For the longitudinal phase, patients were divided into two subgroups (i.e., less atrophy vs. more atrophy) using the median value of each imaging parameter at baseline. Kaplan-Meier curves and log-rank (Mantel-Cox) test were computed to assess the association of baseline imaging variable with the risk of developing each disease milestone and the risk of death over the follow up. For variables significant at the 0.05 level, univariate Cox proportional hazards regression models were computed and HRs and 95% CIs were estimated adjusting for age, disease duration, and phenotype. Statistical significance was set at *p* ≤ 0.05. Data analysis was conducted with SPSS (version 23.0).

## Results

### Demographics, Clinical, and Imaging Features

Demographic, clinical, imaging features, and time to reach disease milestones and death of the 67 PSP patients (52.2% PSP-RS) included in the analysis are shown in Table 1. Irrespective of similar demographics and clinical features, PSP-RS had lower midbrain area and length of midbrain tegmentum and higher pons-to-midbrain diameter ratio compared to vPSP. Clinical phenotypes did not differ in time to reach disease milestones or death.

**Table 1:** Demographic, clinical, imaging features, and time to reach disease milestones and death for the whole cohort and according to disease phenotype.

	**Whole cohort (67)**	**PSP-RS (35)**	**vPSP (32)**	***p***
**Demographics**				
Age, years	70.5 (52–85)	71 (52–79)	70 (56–85)	0.772
Gender (men/women), *n* (%)	32/35 (47.8–52.2)	16/19 (45.71/54.28)	16/16 (50–50)	0.726
**Clinical features**				
Age at onset, years	66 (47–82)	67 (52–79)	65 (47–76)	0.627
Disease duration, years	4 (1–11)	3 (1–11)	5 (1–8)	0.142
PSP rating scale	43 (10–86)	46 (16–86)	42 (10–82)	0.292
**MRI measures**				
Midbrain area, mm^2^	0.74 (0.51–1.31)	0.9 (0.5–1.84)	1.1 (0.63–1.84)	**0.037**
Length of midbrain tegmentum, mm	8.34 (5.07–12.13)	8.01 (5.07–10.63)	9.25 (6.52–12.13)	**0.006**
P_d_/M_d_	2.06 (1.44–2.92)	2.23 (1.45–2.92)	1.94 (1.44–2.67)	**0.006**
Cerebral peduncle angle, degree	68 (52–94)	69 (58–94)	66.5 (52–84)	0.218
MCP/SCP	2.67 (1.27–4.1)	2.67 (1.27–4,1)	2.67 (1.54–3.74)	0.87
P_a_/M_a_	5.68 (2.26–8.96)	6 (2.29–8.96)	5.39 (2.26–8.42)	0.223
MRPI	15.15 (4.2–36.63)	15.82 (4.53–36.63)	13.99 (4.2–31.49)	0.459
P_a_/M_a_ 2.0	1.41 (0.34–2.91)	1.56 (0.57–2,91)	1.28 (0.34–2.63)	0.259
MRPI 2.0	3.74 (0.79–11.89)	4.45 (1.11–11.89)	3.53 (0.79–8.64)	0.393
Frontal volume^*^	134.51 (78.8–161.58)	134.51 (78.8–153.06)	134.22 (107.79–161.58)	0.920
Parietal volume^*^	93.10 (54.26–113.68)	94.14 (54.26–113.68)	91.08 (74.29–107.52)	0.342
Temporal volume^*^	92.26 (35.56–110.49)	93.44 (35.56–110.49)	90.01 (68.75–109.6)	0.585
Occipital volume^*^	43.73 (30.33–54.14)	43.73 (30.33–54.15)	43.55 (36.35–53.45)	0.467
Cingulate volume^*^	16.28 (5.28–20.8)	16.28 (5.28–19.5)	16.39 (13.47–20.8)	0.936
**Disease milestones**				
Time to wheelchair, months	*N* = 30 (44.7%)	*N* = 18 (58.1%)	*N* = 12 (40%)	0.204
	16 (1–42)	16 (1–42)	16 (5–41)	0.871
Time to unintelligible speech, months	*N* = 32 (47.7%)	*N* = 18 (58.1%)	*N* = 14 (48.3%)	0.605
	19 (1–48)	21 (1–48)	18 (5–39)	0.633
Time to dementia, months	*N* = 40 (59.7%)	*N* = 20 (71.4%)	*N* = 20 (69%)	1.000
	16 (1–72)	14 (1–72)	18 (5–38)	0.517
Time to death, months	*N* = 9 (13.4%)	*N* = 7 (24.1%)	*N* = 2 (6.9%)	0.144
	19 (1–72)	21 (1–72)	18 (5–41)	0.641

*Data are expressed in median (range), unless otherwise specified*.

*vPSP included 13 with predominant parkinsonism, 6 with progressive gait freezing, 9 with predominant corticobasal syndrome and 4 with predominant frontal presentation*.

* *Data available for 49 subjects (21 PSP-RS and 28 vPSP)*.

*MCP/SCP, middle cerebellar peduncles to superior cerebellar peduncles ratio; MRPI, MR Parkinsonism index; MRPI 2.0, MR Parkinsonism index 2.0 version; P_a_/M_a_, pons-to midbrain area ratio; P_a_/M_a_ 2.0, pons-to midbrain area ratio 2.0 version; P_d_/M_d_, pons-to-midbrain diameter ratio; PSP-RS, progressive supranuclear palsy with richardson's syndrome; vPSP, the other variant syndromes of Progressive Supranuclear Palsy*.

*Significant differences are highlighted in bold*.

### Association of Imaging Parameters With Disease Severity at Baseline

Imaging correlates of VSGP, severe postural instability and gait impairment are shown in Table 2 and Supplementary Table 1.

**Table 2:** Imaging correlates of vertical supranuclear gaze palsy.

**Variables**	**Univariate *p* value**	**Multivariate OR (CI 95%)**	**Multivariate *p* value**
**Vertical supranuclear gaze palsy**			
Clinical phenotype (PSP-RS vs. vPSP)	0.012	-	NS
Midbrain area	0.003	0.02 (0.00–0.175)	0.006
Pons area	0.120	-	-
P_a_/M_a_	0.022	-	Not included
MCP	0.085	-	NS
SCP	0.091	-	NS
MCP/SCP	0.462	-	-
MRPI	0.076	-	Not included
Third ventricle width	0.772	-	-
Frontal horns width	0.965	-	-
P_a_/M_a_ 2.0	0.125	-	-
MRPI 2.0	0.175	-	-
Interpeduncolar angle	0.117	-	-
Pons diameter	0.012	-	NS
Midbrain diameter	0.012	-	NS
P_d_/M_d_	0.016	-	Not included
Lenght of midbrain tegmentum	0.004	-	NS
Frontal volume	0.140	-	-
Parietal volume	0.960	-	-
Temporal volume	0.662	-	-
Occipital volume	0.065	-	NS
Cingulate volume	0.252	-	-

*Each univariate as well as multivariate models were adjusted for age and disease duration*.

*The midbrain ratios were only considered in the multivariate model if neither of the individual components was significant*.

*M_d_, Midbrain diameter; MCP/SCP, middle cerebellar peduncles to superior cerebellar peduncles ratio; MRPI, MR Parkinsonism index; MRPI 2.0, MR Parkinsonism index 2.0 version; NS, not significant; OR, odds ratio; P_a_/M_a_, pons-to midbrain area ratio; P_a_/M_a_ 2.0, pons-to midbrain area ratio 2.0 version; P_d_/M_d_, pons-to-midbrain diameter ratio; PSP-RS, progressive supranuclear palsy with Richardson's syndrome; vPSP, the other variant syndromes of progressive supranuclear palsy*.

After adjusting for age and disease duration in the univariate model, VSGP was associated with PSP-RS phenotype, midbrain area, midbrain diameter, pons diameter, length of midbrain tegmentum, MCP, SCP, MRPI, P/M, pons-to-midbrain diameter ratio, occipital volume (all *p* < 0.1). Only association with smaller midbrain area was confirmed in the multivariate model (Table 2).

After adjusting for age and disease duration in the univariate model, severe postural instability was associated with PSP-RS phenotype, midbrain area, midbrain diameter, pons-to-midbrain diameter ratio, M/P, M/P 2.0, MRPI, MRPI 2.0, length of midbrain tegmentum, MCP/SCP, third ventricle and frontal horns width and cingulate volume (all *p* < 0.1). In the multivariate model, all became non-significant (Supplementary Table 1).

After adjusting for age and disease duration in the univariate model, severe gait impairment was associated with PSP-RS phenotype, midbrain area, midbrain diameter, SCP, pons diameter and M/P (all *p* < 0.1). In the multivariate model, all became non-significant (Supplementary Table 1).

### Association of Imaging Parameters at Baseline With Disease Progression

**Figure 2:**
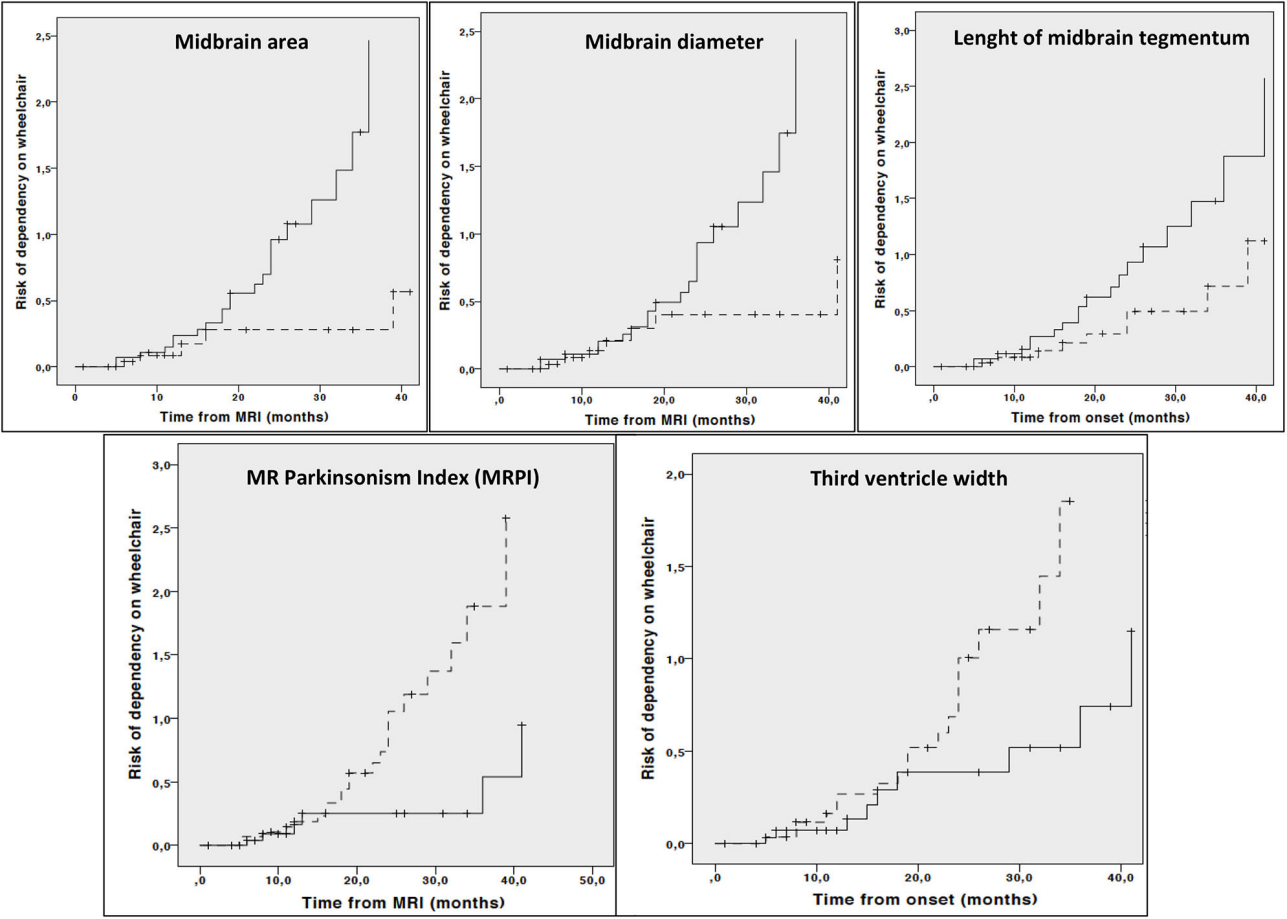
Cumulative risk of dependency on wheelchair by each MRI parameter (less atrophy vs. more atrophy). Only curves with significant log-rank test at 0.05 level are reported. Dotted line: values above median; Continuous line: values below median.

**Figure 3:**
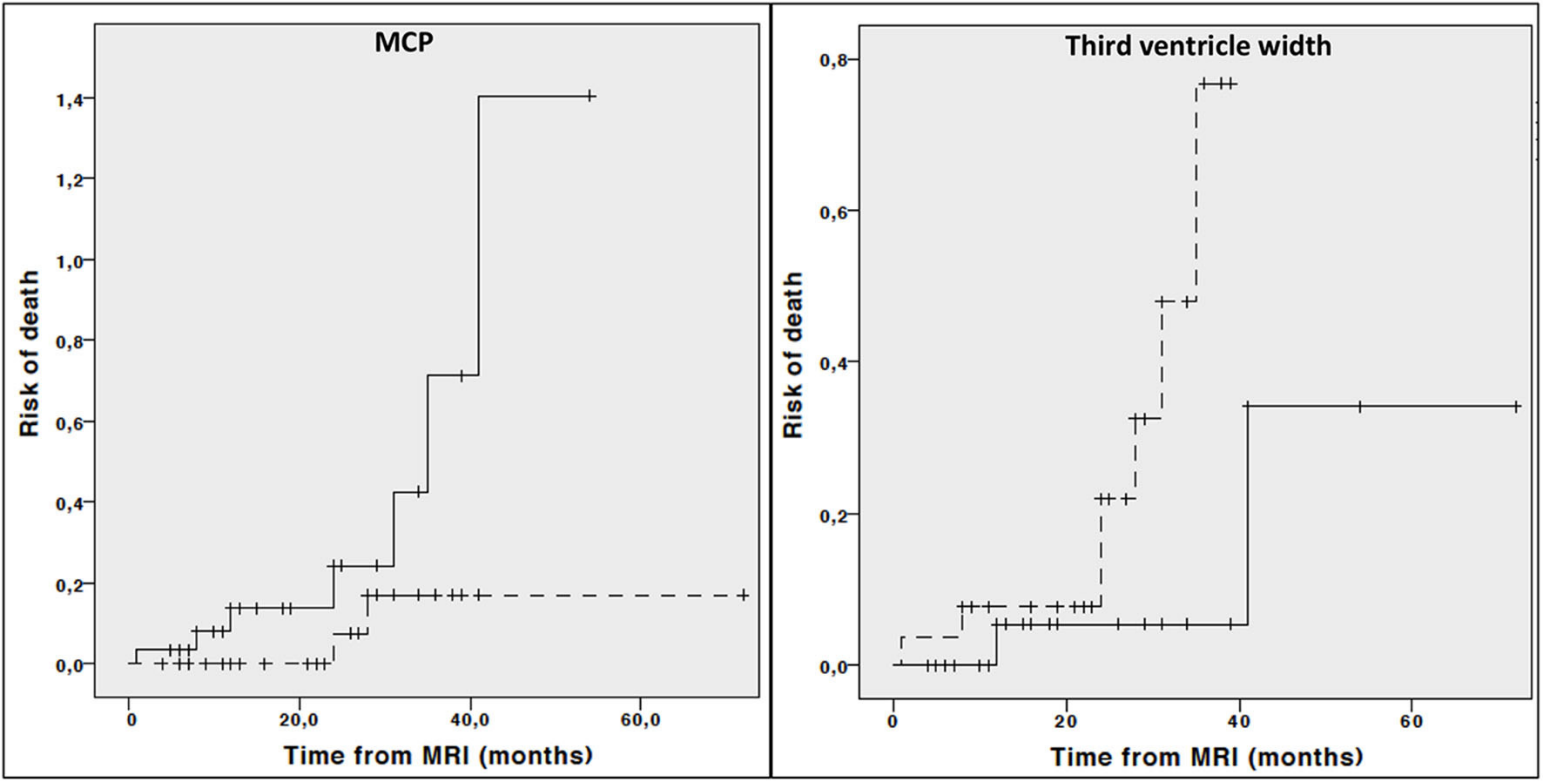
Cumulative risk of death by each MRI parameter (less atrophy vs. more atrophy). Only curves with significant log-rank test at 0.05 level are reported. Dotted line: values above median; Continous line: values below median.

Kaplan Meier curves showed lower midbrain area (*p* = 0.006), midbrain diameter (*p* = 0.013), length of midbrain tegmentum (*p* = 0.044), higher MRPI (*p* = 0.008) and larger third ventricle width (*p* = 0.029) were associated with higher risk of dependency on wheelchair (Figure 2). In addition, lower MCP (*p* = 0.044) and larger third ventricle width (*p* = 0.050) were associated with higher risk of death (Figure 3).

Cox regression analysis adjusted for age, disease duration and phenotype demonstrated that lower midbrain area (HR: 0.122, 95% CI 0.030–0.493, *p* = 0.003), midbrain diameter (HR: 0.313, 95% CI 0.112–0.878, *p* = 0.027), higher MRPI (HR: 6.162, 95% CI 1.790–21.209, *p* = 0.004) and larger third ventricle width (HR: 2.755, 95% CI 1.068–7.108, *p* = 0.036) but not length of midbrain tegmentum (HR: 0.431, 95% CI 0.176–1.056, *p* = 0.066) were associated with a significant higher risk of dependency on wheelchair. On the contrary, neither third ventricle width (HR: 3.328 95% CI 0.358–30.997, *p* = 0.291) or MCP (HR: 0.263, 95% CI 0.047–1.467, *p* = 0.128) were associated with higher risk of death when considering age, disease duration and phenotype as covariate.

## Discussion

Although previous studies have shown association of regional brain atrophy with disease features in PSP (8, 9, 28, 29), this is the first comprehensive study to systematically assess the association of available midbrain-based MRI measures and cortical volumes with disease severity and progression in a large cohort of patients with PSP in a real-world setting. Our findings showed that, irrespective to other disease and imaging features, reduced midbrain area is significantly associated with greater ocular motor dysfunction at the time of MRI and more rapid disease progression on follow up.

In the cross-sectional part of our study, we demonstrated a direct association between VSGP and midbrain area. This finding was not surprising as a large body of evidence confirms that vertical saccades are triggered by the rostral interstitial nucleus of the medial longitudinal fasciculus located in the midbrain (30). As such, vertical saccades as assessed with video-oculography have been linked with midbrain size as well as midbrain network dysfunction in PSP (28, 29). Our data are also in line with previous evidence of reduced midbrain area in patients with VSGP (O1 domain from MDS criteria) compared to those with slowing of vertical saccades (O2 domain) (31).

Given our methodological approach, we were able to show that ocular motor dysfunction is specifically linked to the midbrain, that is, a brain region crucially involved in the underlying PSP-related pathological process (2, 32). Several methodological considerations strengthen our data. First, the statistical approach provided direct evidence of such relationship irrespective of age, disease duration, and phenotype as well as other MRI parameters (28, 29). Second, clinical evaluation of ocular movements was rated with specific items from the PSP rating scale, the most used tool in the context of disease-modifying clinical trials, with no need to use more sophisticated and time-consuming assessments (13). Finally, our patients were diagnosed and phenotyped with the most up-to-date set of available criteria (1).

On the other hand, relationship between gait impairment and postural instability with multiple midbrain parameters (area, diameter, length of tegmentum) was demonstrated in the univariate analysis only and not confirmed when accounting for age, disease duration and phenotype as well as other MRI parameters. Taken together such findings suggest that a broader disruption of the complex interplay between different networks and structures likely underpins the severity of gait and postural issues in PSP (29).

In the longitudinal part of our study, the relationship between multiple baseline midbrain-based parameters as well as cortical lobe volumes and disease-specific milestones was investigated. Again, irrespective of age, disease duration, and phenotype, different direct and indirect midbrain parameters (i.e, midbrain area, midbrain diameter, third ventricle width and MRPI) predicted higher risk of dependency on wheelchair. On the other hand, relationship between risk of death with MCP and third ventricle width was demonstrated in the univariate analysis only and not confirmed when accounting for age, disease duration and phenotype. Finally, none of baseline MRI measures were able to predict either dementia or unintelligible speech or survival.

In line with previous data (8), our findings suggest midbrain area—the simplest imaging parameter to measure—is able to predict disease progression. This adds to data from a previous study showing a relationship between midbrain tau-PET signal and severity of disease as assessed with PSP rating scale (33). From a practical point of view, midbrain area measurement is much easier to perform by neuroradiologists and general radiologists not specifically working with movement disorders (it requires only the mid-sagittal image without further specific reconstructed planes or repetitive and less reproducible measurements on the superior and middle cerebellar peduncles) and, thus, might be more appropriate than other measures in a real-world setting.

Strikingly, we failed to find any relationship between cortical lobe volumes and development of dementia. A note of caution on such conclusion, however, is that cortical lobe volumes were available only for a limited number of subjects (i.e., 49/67). Also, dementia was evaluated according to clinician-based opinion instead of using a formal cognitive battery. Similarly, lack of any association with risk of death may be related to the lower percentage of deceased in our cohort (13.4%) over the follow up. Finally, we acknowledge we used a simplistic approach considering cortical lobe volumes and, as such, we can not exclude specific cortical regions would be associated with impairment in specific functional domains. However, this was out of the scope of the present analysis.

Our study has limitations. First, we recognize the lack of pathological confirmation of both diagnosis and phenotypic categorization, still the gold standard for PSP diagnosis. Although our data are based only on clinical judgment, both the MDS diagnostic flow chart and phenotypic attribution have been applied independently by two experts in movement disorders as detailed elsewhere (7). In addition, evaluation of clinical features was conducted by movement disorders specialists with more than 10 years of experience in movement disorders (MP, RC and DF).

As a second drawback, we acknowledge that merging PSP with predominant parkinsonism, PSP with progressive gait freezing, PSP with predominant corticobasal syndrome and PSP with predominant frontal presentation into a single group (i.e., vPSP) to increase the statistical power of our analysis may limit the interpretation of the results for PSP subtype comparisons. Indeed, further studies enrolling a larger number of phenotypes other than PSP-RS are needed to better characterize the relationship between radiological biomarkers and disease severity and progression according to phenotype. Also we failed to report on other important clinical milestones as dysphagia.

From a technical standpoint, we recognize the lack of longitudinal MRIs from a healthy control group prevent us from quantifying atrophy rate in PSP compared to age-matched healthy subjects. However, previous data already shown midbrain area had diagnostic value in differentiating PSP vs. healthy controls and prognostic value as marker of disease progression in PSP (10, 34, 35). Furthermore, we recognize MRI imaging was not standardized across the two sites. However, 82% of the cohort was enrolled at Salerno center and underwent MRI with the same facility. Also, midbrain-based morphometric assessments were computed by the same examiner (RM) who received the whole set of images and personally performed sagittal partitions and multiplanar reconstructions. Finally, cortical volumes were available for a subset of 49 patients, all from the center of Salerno.

In conclusion, our study demonstrates that, irrespective of disease features and other MRI parameters, reduced midbrain size is significantly associated with greater ocular motor dysfunction at the time of MRI and more rapid disease progression on follow up. Longitudinal imaging studies are required to validate midbrain area as a marker of disease progression in different phenotypes and accounting for other disease and imaging features.

This is the first comprehensive study to systematically assess the association of available midbrain-based MRI measures and cortical lobe volumes with disease severity and progression in a large cohort of patients with PSP diagnosed according with MDS criteria in a real-world setting.

## Data Availability Statement

The deidentified dataset used to generate the results in the current study is available from the corresponding author on request.

## Ethics Statement

The studies involving human participants were reviewed and approved by Ethic Committee Campania Tre Sud. The patients/participants provided their written informed consent to participate in this study.

## Author Contributions

MP: conception, organization, and execution of the research project, design and execution of statistical analysis, and writing the first draft of the manuscript. FA, SP, and MT: organization and execution of the research project, review and critique of statistical analysis, and review and critique of the manuscript. RE, ED, PC, MC, RC, GS, FS, FE, MTP, RM, and PB: execution of the research project, review and critique of statistical analysis, and review and critique of the manuscript. All authors contributed to the article and approved the submitted version.

## Conflict of Interest

The authors declare that the research was conducted in the absence of any commercial or financial relationships that could be construed as a potential conflict of interest.

## Abbreviations

CI: confidence interval
HR: hazard ratio
MCP/SCP: middle cerebellar peduncles to superior cerebellar peduncles ratio
MDS: movement disorder society
MRI: magnetic resonance imaging
MRPI: MR Parkinsonism index
MRPI 2.0: MR Parkinsonism index 2.0 version
OR: odds ratio
P/M: pons-to-midbrain area ratio
P/M 2.0: pons-to-midbrain area ratio 2.0 version
PSP: progressive supranuclear palsy
PSP-RS: PSP Richardson's syndrome
vPSP: other variant syndromes of PSP
VSGP: vertical supranuclear gaze palsy.
