# Supplementary material for: Association of MRI Measures With Disease Severity and Progression in Progressive Supranuclear Palsy
Source: Front Neurol. 2020 Nov 12;11:603161. doi: 10.3389/fneur.2020.603161 (PMC7688910; doi:10.3389/fneur.2020.603161)
Supplement: Supplementary file 1 [file Table_1.docx]

**Table S1. Imaging correlates of severe postural instability and gait impairment**

| **Variables** | **Univariate**  **p value** | **Multivariate**  **OR (CI 95%)** | **Multivariate**  **P value** | **Univariate**  **p value** | **Multivariate**  **OR (CI 95%)** | **Multivariate**  **P value** |
| --- | --- | --- | --- | --- | --- | --- |
|  | **Severe postural instability** | | | **Severe gait impairment** | | |
| **Clinical phenotype (PSP-RS vs vPSP)** | 0.021 | - | NS | 0.053 | - | NS |
| **Midbrain area** | 0.017 | - | NS | 0.045 | - | NS |
| **Pons area** | 0.771 | - | - | 0.327 | - | - |
| **P/M** | 0.003 | - | Not included | 0.077 | - | Not included |
| **MCP** | 0.477 | - | - | 0.110 | - | - |
| **SCP** | 0.126 | - | - | 0.066 | - | NS |
| **MCP/SCP** | 0.059 | - | NS | 0.321 | - | - |
| **MRPI** | 0.002 | - | - | 0.102 | - | - |
| **Third ventricle width** | 0.036 | - | NS | 0.912 | - | - |
| **Frontal horns width** | 0.058 | - | NS | 0.222 |  |  |
| **P/M 2.0** | 0.002 | - | Not included | 0.447 | - | - |
| **MRPI 2.0** | 0.002 | - | Not included | 0.376 | - | - |
| **Interpeduncolar angle** | 0.127 | - | - | 0.336 | - | - |
| **Pons diameter** | 0.926 | - | - | 0.036 | - | NS |
| **Midbrain diameter** | 0.004 | - | NS | 0.058 | - | NS |
| **Pons-to-midbrain diameter ratio** | 0.007 | - | NS | 0.122 | - | - |
| **Length of midbrain tegmentum** | 0.012 | - | NS | 0.112 | - | - |
| **Frontal volume** | 0.227 | - | - | 0.959 | - | - |
| **Parietal volume** | 0.203 | - | - | 0.770 | - | - |
| **Temporal volume** | 0.158 | - | - | 0.821 | - | - |
| **Occipital volume** | 0.173 | - | - | 0.301 | - | - |
| **Cingulate volume** | 0.080 | - | NS | 0.293 | - | - |

Each univariate as well as multivariate models were adjusted for age and disease duration.

The midbrain ratios were only considered in the multivariate model if neither of the individual components was significant.

Abbreviations: MCP/SCP: middle cerebellar peduncles to superior cerebellar peduncles ratio; MRPI: MR Parkinsonism Index; MRPI 2.0: MR Parkinsonism Index 2.0 version; NS: not significant; OR: odds ratio; P/M: pons-to midbrain area ratio; P/M 2.0: pons-to midbrain area ratio 2.0 version; PSP-RS: Progressive Supranuclear palsy with Richardson’s syndrome; vPSP: the other variant syndromes of Progressive Supranuclear Palsy.
